# Quantitative Analysis of *Psoralea corylifolia* Linne and its Neuroprotective and Anti-Neuroinflammatory Effects in HT22 Hippocampal Cells and BV-2 Microglia

**DOI:** 10.3390/molecules21081076

**Published:** 2016-08-17

**Authors:** Yu Jin Kim, Hye-Sun Lim, Jun Lee, Soo-Jin Jeong

**Affiliations:** 1 Herbal Medicine Research Division, Korea Institute of Oriental Medicine, Daejeon 34054, Korea; jinjin0228@kiom.re.kr (Y.J.K.); qp1015@kiom.re.kr (H.-S.L.); junlee@kiom.re.kr (J.L.); 2 Korean Medicine Life Science, University of Science & Technology, Daejeon 34113, Korea

**Keywords:** *Psoraleae corylifolia*, bakuchiol, quantitative analysis, neuroprotection, neuro-inflammation

## Abstract

The seeds of *Psoralea corylifolia* L. (*P. corylifolia*), also known as “Bo-Gol-Zhee” in Korea, are used in a traditional herbal medicine for treating various skin diseases. In the present study, we performed quantitative analyses of the seven standard components of *P. corylifolia*: psoralen, angelicin, neobavaisoflavone, psoralidin, isobavachalcone, bavachinin, and bakuchiol, using high-performance liquid chromatography. We also investigated the neuroprotective and anti-neuroinflammation effects of *P. corylifolia* and its standard components in the hippocampal cell line HT22 and microglia cell line BV-2. A 70% ethanol extract of *P. corylifolia* was prepared and the seven standard components were separated using C-18 analytical columns by gradient solvents with acetonitrile and water, and ultraviolet detection at 215, 225 and 275 nm. The analytical method showed high linearity, with a correlation coefficient of ≥0.9999. The amounts of the standard components ranged from 0.74 to 11.71 mg/g. Among the components, bakuchiol (11.71 mg/g) was the most potent phytochemical component of *P. corylifolia*. Furthermore, we analyzed the inhibitory effects of the components from *P. corylifolia* to determine the bioactive compound needed to regulate neuronal cell changes. Angelicin, isobavachalcone, and bakuchiol suppressed lipopolysaccharide (LPS)-stimulated nitric oxide production in LPS-treated BV-2 microglia more significantly than did the other components. In HT22 hippocampal cells, neobavaisoflavone and bakuchiol had more potent inhibitory activity against hydrogen peroxide-induced cell death. Taken together of the quantification and efficacy analyses, bakuchiol appeared to be the most potent bioactive phytochemical component of *P. corylifolia* for the potential treatment of neurodegenerative diseases.

## 1. Introduction

**Figure 1:**
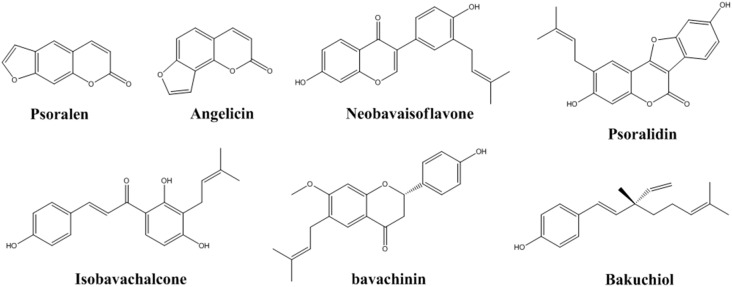
Chemical structures of the seven marker compounds of *P. corylifolia*.

The seeds of *Psoralea corylifolia* L. (*P. corylifolia*), also called “Bo-Gol-Zhee” in Korean and Buguzhi in Chinese, are used in a traditional herbal medicine. *P. corylifolia* belongs to the genus *Psoralea* and the seeds have been used widely for the treatment of various types of skin diseases such as vitiligo, alopecia areata, leukoderma, and psoriasis [1]. To date, over 90 compounds have been identified from *P. corylifolia*. Among them, the seven major compounds are psoralen, angelicin, neobavaisoflavone, psoralidin, isobavachalcone, bavachinin, and bakuchiol (Figure 1), which were reported as biologically active standard components [2]. *P. corylifolia* and its standard components are known to have medicinal properties in combating diabetes [3,4], obesity [5], tumorigenesis [6,7], oxidative stress [4,8], and inflammation [9,10], and to have estrogen-like effects [11,12]. Several groups have reported the possibility of using *P. corylifolia* or its major components as drug(s) for treating neurodegenerative diseases or depression [13,14,15]. However, no report has determined the active components of *P. corylifolia* against crucial cellular changes such as neuroinflammation and neuronal cell damage. Our present study investigated the biological activity of seven components of *P. corylifolia* in the prevention of neuroinflammation in BV-2 microglia and the neuroprotection of HT22 hippocampal cells.

## 2. Results

### 2.1. Optimization of High-Performance Liquid Chromatography (HPLC) Separation

**Figure 2:**
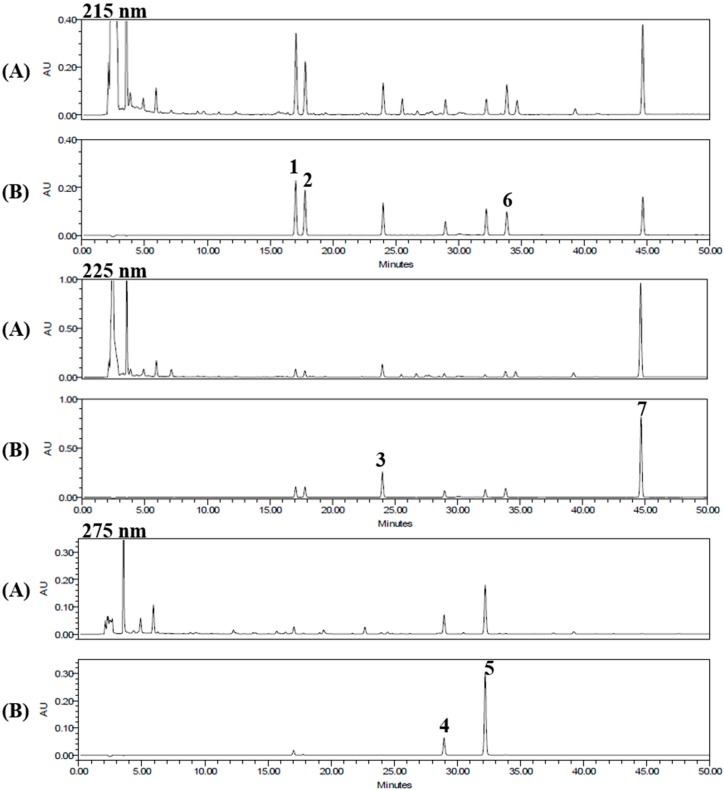
HPLC chromatograms of the 70% ethanol extract of *P. corylifolia* seeds (**A**); and its standard mixture (**B**) at 215 nm, 225 nm, and 275 nm. Psoralen (**1**), angelicin (**2**), neobavaisoflavone (**3**), psoralidin (**4**), isobavachalcone (**5**), bavachinin (**6**), and bakuchiol (**7**).

We used HPLC for separation of the seven standard components from the 70% ethanol extract of the seeds of *P. corylifolia*. The established conditions of the mobile phase was shown in Table 1. Under these established HPLC methods, the seven standard components were resolved within 45 min. The retention times of the psoralen, angelicin, neobavaisoflavone, psoralidin, isobavachalcone, bavachinin, and bakuchiol were 17.05, 17.79, 23.99, 28.95, 32.21, 33.84, and 44.65 min, respectively. HPLC chromatograms of the 70% ethanol extract of the seeds of *P. corylifolia* and the standard mixture are shown in Figure 2.

### 2.2. Linearity, Limits of Detection (LOD), and Limits of Quantification (LOQ)

The linear relationships between the peak areas (*y*) and concentrations (*x*, μg/mL) of the components were expressed by the regression equations (*y* = a*x* + b) given in Table 2. The established analytical method showed high linearity with a correlation coefficient (*r*^2^) of ≥0.9999. The calibration curves showed good linearity over the concentration range 3.125–100 μg/mL, except for bakuchiol (12.5–400 μg/mL). The LODs and LOQs for the seven standard components were in the range 0.102–0.988 μg/mL and 0.309–2.995 μg/mL, respectively.

### 2.3. Determination of the Seven Standard Components in P. corylifolia

The established HPLC analytical method was applied to the simultaneous quantification of the seven components in the seed of *P. corylifolia*. The amounts of the seven standard components ranged from 0.74 mg/g to 11.71 mg/g. Among the components, bakuchiol was the most abundant compound in the seed of *P. corylifolia*. The results for the content of each component are shown in Table 3.

### 2.4. Anti-Neuroinflammatory Effects of Seven Standard Components in P. corylifolia

**Figure 3:**
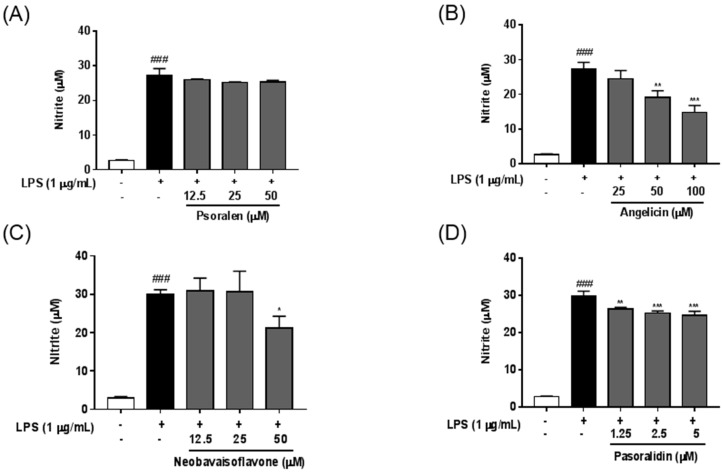

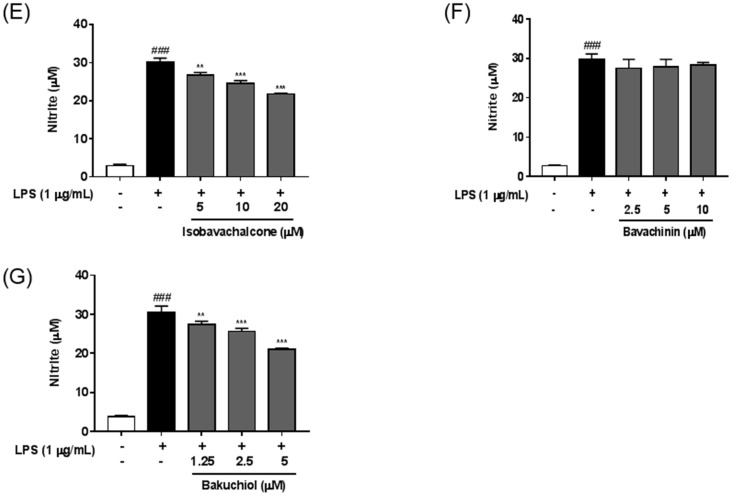
Anti-neuroinflammatory effects of the seven components from *P. corylifolia* in LPS-stimulated BV-2 cells. Cells were pretreated with various concentrations of each components for 2 h and then stimulated with LPS (1 μg/mL) for an additional 22 h. Results are shown for psoralen (**A**); angelicin (**B**); neobavaisoflavone (**C**); psoralidin (**D**); isobavachalcone (**E**); bavachinin (**F**); and bakuchiol (**G**). The production of NO was determined using Griess reagents. The results are expressed as the mean ± SEM of three independent experiments. ^###^ *p* < 0.01 versus vehicle control cells; * *p* < 0.05, ** *p* < 0.01, and *** *p* < 0.001 versus LPS-treated cells.

We evaluated the effect of the seven standard components of *P. corylifolia* on LPS-induced nitric oxide (NO) production in BV-2 microglia cells. As shown in Figure 3, LPS stimulation strongly increased NO production in BV-2 cells compared with untreated controls and, treatment with LPS (1 μg/mL) did not induce obvious decrease of cell viability, suggesting that LPS were non-toxic to microglia cell during this concentration range. Among these components, angelicin, isobavachalcone, and bakuchiol had the most significant inhibitory effect on the LPS-induced NO production in dose-dependent manners. Psoralidin also significantly suppressed LPS-induced NO production in BV-2 cells. Neobavaisoflavone had an inhibitory effect only at 50 μM. These effects were not caused by cytotoxicity because components at these concentrations did not show any significant reductions in cell viability.

### 2.5. Neuroprotective Effects of Seven Standard Compounds in P. corylifolia

**Figure 4:**
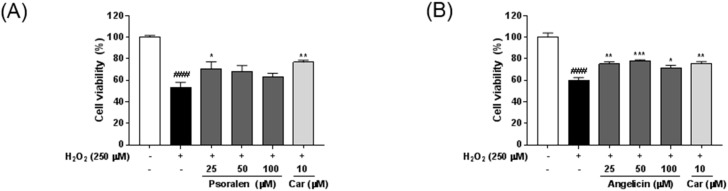

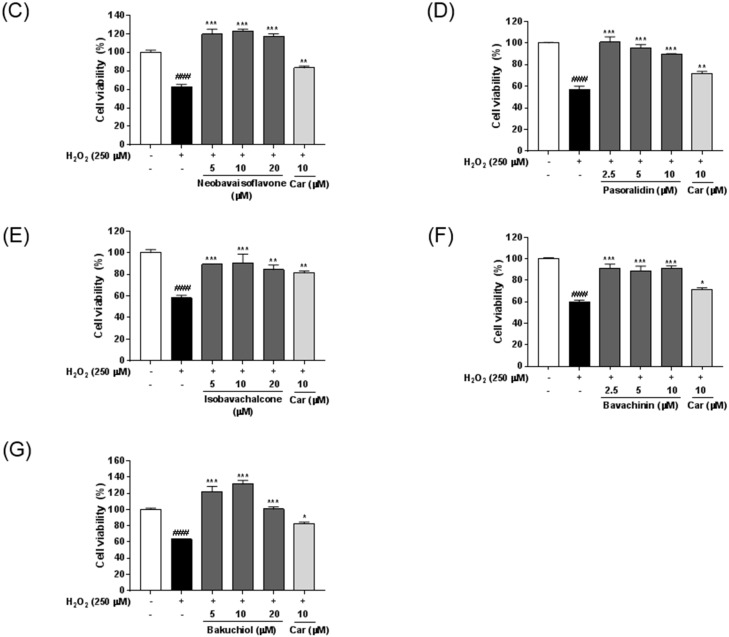
Neuroprotective effects of the seven components from *P. corylifolia* in H_2_O_2_-treated HT22 cells. Cells were cotreated with various concentrations of each components and H_2_O_2_ (250 μM) for 6 h. Psoralen (**A**); angelicin (**B**); neobavaisoflavone (**C**); psoralidin (**D**); isobavachalcone (**E**); bavachinin (**F**); and bakuchiol (**G**). Cell viability was assessed using CCK-8 assays. Carvedilol (Car) was used as a positive control. The results are expressed as mean ± SEM of three independent experiments. ^###^ *p* < 0.01 versus vehicle control cells; * *p* < 0.05, ** *p* < 0.01, and *** *p* < 0.001 versus H_2_O_2_-treated cells.

To investigate whether the seven standard components of *P. corylifolia* acted against neuronal cell damage, HT22 mouse hippocampal cells were treated with hydrogen peroxide (H_2_O_2_) in the presence or absence of various concentrations of each component. Cytotoxicity of seven standard components was determined using CCK assay (Supplementary Figure S1), and nontoxic concentrations of each component were used for the following experiments. As shown in Figure 4, H_2_O_2_ treatment significantly reduced the viability of HT22 cells compared with untreated controls. All the standard components exerted protective effects against H_2_O_2_-induced neuronal cell damage. Psoralen weakly reversed H_2_O_2_-induced neuronal cell death, but only at 25 μM. Neobavaisoflavone and bakuchiol had the most significant inhibitory effects against H_2_O_2_-induced damage. Carvedilol was used as a positive control [16].

## 3. Discussion

Neuroinflammation plays an important role in the pathogenesis of various neurodegenerative diseases such as Alzheimer’s disease and other dementias, Parkinson’s disease, and Huntington’s disease [17]. Microglia act as key mediators of neuroinflammation although they represent only about 10% of the total cell population in the central nervous system [18,19]. Activated microglia generate proinflammatory cytokines such as tumor necrosis factor-alpha, interleukin (IL)-1, and IL-6 and trigger the production of NO [20]. Excessive NO production stimulates the generation of reactive nitrogen species and mediates neuronal cell death [21,22]. Thus, the targeting NO production is thought to be a valuable clinical approach for the treatment of neurodegenerative diseases [23].

In neurodegenerative diseases, H_2_O_2_ is one of the most important mediators of oxidative stress detected under pathological conditions. H_2_O_2_ generation is required to mediate the complete sequence of events occurring in oxidative stress-induced neuronal cell death [24]. In previous studies, researchers employed H_2_O_2_ overload as a neurotoxic challenge paradigm to evaluate aspects of neuroprotection in murine hippocampal HT22 cell [25,26].

In the present study, we examined whether the seven components influenced the production of NO in LPS-stimulated BV-2 microglia. Among the components, angelicin, psoralidin, iso-bavachalcone, neobavaisoflavone and bakuchiol significantly decreased the LPS-induced NO production in BV-2 cells. Neobavaisoflavone inhibited the NO production only at higher concentration (50 μM) and psoralidin showed mild effects on the NO inhibition compared with the other components. Angelicin, isobavachalcone, and bakuchiol reduced the NO levels in dose-dependent manners in LPS-treated BV-2 cells. We also investigated the inhibitory effects of *P. corylifolia* and its seven components using HT22 hippocampal cells damaged by H_2_O_2_. All tested compounds revealed their potential as neuroprotective agents. However, psoralen and angelicin had weaker inhibitory activities than the others. Neobavaisoflavone and bakuchiol most significantly inhibited the H_2_O_2_-induced death of HT22 cells. In quantification of the seven components from *P. corylifolia*, bakuchiol was the most prevalent (11.71 mg/g) compared with the other six components. Overall, considering the quantification and bio-efficacy analyses, bakuchiol proved the most potent bioactive phytochemical of *P. corylifolia* for the potential treatment of neurodegenerative diseases.

Bakuchiol from *P. corylifolia* has a variety of biological activities such as inhibiting tumorigenesis [27,28], fungal activity [29], viral infections [30], bone loss [31], and hepatotoxicity [32], and has estrogenic-like effects [33]. Of note, Chaudhuri et al. reported that bakuchiol can act as an anti-aging compound via regulation of retinol-like gene expression [34]. Taken together, we consider that bakuchiol might be more useful for treating age-associated neurodegenerative diseases such as Alzheimer’s disease. Further studies will be necessary to understand the molecular mechanisms responsible for the regulation of neuronal cell responses using in vitro and in vivo experimental models of Alzheimer’s disease. In addition, the safety of bakuchiol should be elucidated by toxicology testing.

## 4. Materials and Methods

### 4.1. Plant Material

Seeds of *Psoralea corylifolia* were purchased from the Kwangmyungdang herbal market (Ulsan, Korea). A voucher specimen has been deposited at the Herbal Medicine Research Division, Korea Institute of Oriental Medicine.

### 4.2. Chemicals and Reagents

The standard components, psoralen, angelicin, neobavaisoflavone, psoralidin, isobavachalcone, bavachinin, and bakuchiol were purchased from Shanghai Sunny Biotech Co., Ltd. (Shanghai, China). The chemical structures of the standard components were shown in Figure 1. The purities of these standard components were ≥98.0% by high-performance liquid chromatography (HPLC) analysis. The HPLC-grade solvents, acetonitrile and water, were obtained from J. T. Baker Chemical Co. (Phillipsburg, NJ, USA).

### 4.3. Apparatus and Chromatographic Conditions

Quantitative analysis was conducted using a Waters Alliance e2695 system (Waters Corp., Milford, MA, USA) equipped with a pump, degasser, column oven, autosampler, and photodiode array detector (Waters Corp., #2998). The data were acquired and processed using Empower software (version 3; Waters Corp). Chromatographic separation for the seven standard components was carried out at room temperature using Luna C_18_ analytical columns (250 mm × 4.6 mm, 5 μm) supplied by Phenomenex (Torrance, CA, USA) with a gradient solvent system of acetonitrile and water. The ultraviolet (UV) wavelengths for detecting components were 215 nm for psoralen, angelicin and bavachinin; 225 nm for neobavaisoflavone and bakuchiol; and 275 nm for psoralidin and isobavachalcone. The flow rate was 1.0 mL/min and the injection volume was 10 μL.

### 4.4. Preparation of Standard Solutions

The seven components were weighed accurately, dissolved in methanol at 1.0 mg/mL and stored at below 4 °C. The stock solutions were diluted to yield a series of standard solutions with different concentrations for quantitative analysis.

### 4.5. Preparation of Sample Solutions

Dried seeds of *P. corylifolia* (50 g) were extracted twice with 70% ethanol (300 mL) by refluxing for 2 h. The extracted solution was filtered through a filter paper (5 μm), and evaporated using a rotary evaporator under a vacuum to dryness (8.279 g). The 70% ethanol extract of the seeds of *P. corylifolia* was weighed accurately and dissolved in methanol at 20 mg/mL. The sample solution was filtered through a syringe filter (0.45 μm) for HPLC analysis.

### 4.6. Calibration Curve and Determination of the Limit of Detection (LOD) and Limit of Quantification (LOQ)

The calibration curves of components were obtained by assessment of the peak areas of the standard solutions at six different concentrations. The tested concentration ranges were 3.125–100 μg/mL for psoralen, angelicin, neobavaisoflavone, psoralidin, isobavachalcone, and bavachinin, and 12.5–400 μg/mL for bakuchiol. The LOD and LOQ for the seven standard components were calculated using the slope of the calibration curve and the standard deviation (SD) of the intercept as follows: LOD = 3.3 × (SD of the response/slope of the calibration curve); and LOQ = 10 × (SD of the response/slope of the calibration curve).

### 4.7. Cell Lines and Culture

Mouse microglia BV-2 is a cell lines generated by infecting with a v-raf/v-myc oncogene carrying retrovirus in primary microglial cell [35]. Mouse hippocampal HT22 is a cell line immortalized a subclone of the original clone HT4 [36]. The murine microglial cell line BV2 and hippocampal HT22 cells were purchased from the American Type Culture Collection (Manassas, VA, USA) and cultured in Dulbecco’s Modified Eagle’s medium (Hyclone/Thermo Fisher Scientific, Rockford, IL, USA), supplemented with 10% fetal bovine serum (Hyclone/Thermo Fisher) and penicillin/streptomycin under 5% CO_2_ in air at 37 °C.

### 4.8. Nitric Oxide (NO) Assay

NO synthesis was analyzed by determining the accumulation of nitrite (NO_2_^−^) in culture supernatants using the Griess Reagent System (Promega, Madison, WI, USA). BV-2 cells were pretreated with seven standard components for 2 h and treated with lipopolysaccharide (LPS; 1 μg/mL, Sigma-Aldrich, St. Louis, MO, USA) for an additional 22 h. After collecting the culture supernatants, equal volumes of supernatant and sulfanilamide solution were mixed, incubated for 10 min at room temperature, and then added to naphthylethylenediamine dihydrochloride solution for an additional 5 min. The absorbance was measured at 540 nm by using an Epoch microplate spectrophotometer. The nitrite concentration was determined from a standard curve (100, 50, 25, 12.5, 6.25, 3.13, 1.56 μM) generated using sodium nitrite (NaNO_2_) solutions. Determine average absorbance value of each experimental sample. Determine its concentration by comparison to the standard curve.

### 4.9. Measurement of Neuroprotective Activity

HT22 cells were plated on 96-well microplates at a density of 5 × 10^3^/well and co-treated with hydrogen peroxide (H_2_O_2_, 250 μM, Sigma-Aldrich) and various concentrations of each marker compound for 6 h. Cell counting Kit-8 (CCK-8) solution (Dojindo, Kumamoto, Japan) was added, and the cells were incubated for 4 h. The absorbance was read at 450 nm on an Epoch Microplate Spectrophotometer (BioTek Instruments, Inc., Winooski, VT, USA). The cell viability was calculated using the following equation:

$$\text{Cell viability }\left(\text{\%}\right)=\frac{\text{Mean OD in drug}-\text{treated cells}}{\text{Mean OD in untreated cells}}\times 100$$

### 4.10. Statistical Analysis

The data are expressed as the mean ± standard error of the mean (SEM). Data were analyzed using one-way analysis of variance and Dunnett’s multiple comparisons test; *p* < 0.05 was considered statistically significant.

## Figures and Tables

**Table 1:** Condition of mobile phase for HPLC analysis.

Time (min)	Flow Rate (mL/min)	Mobile Phase	
		Water (%)	Acetonitrile (%)
0	1.0	75	25
40	1.0	20	80
46	1.0	0	100
52	1.0	0	100

**Table 2:** Linear range, regression equation, correlation coefficients, LODs, and LOQs for compounds.

Compound	Linear Range (μg/mL)	Regression Equation (*y* = a*x* + b) ^a^		Correlation Coefficient (*r*^2^)	LOD ^b ^(μg/mL)	LOQ ^c ^(μg/mL)
		Slope (a)	Intercept (b)			
Psoralen	3.125–100	81466	73697	0.9999	0.102	0.309
Angelicin	3.125–100	68433	61942	0.9999	0.103	0.313
Neobavaisoflavone	3.125–100	46488	25575	1.0000	0.239	0.725
Psoralidin	3.125–100	24213	1833.9	1.0000	0.134	0.407
Isobavachalcone	3.125–100	113946	60594	1.0000	0.175	0.529
Bavachinin	3.125–100	39030	33550	0.9999	0.190	0.576
Bakuchiol	12.5–400	38481	−29888	1.0000	0.988	2.995

**^a^** *y* = a*x* + b, *y* means peak area and *x* means concentration (μg/mL); **^b ^** LOD (Limit of detection): 3.3 × (SD of the response/slope of the calibration curve); **^c ^** LOQ (Limit of quantitation): 10 × (SD of the response/slope of the calibration curve).

**Table 3:** The content of maker compounds in the seed of *Psoralea corylifolia* L.

Compound	Content (mg/g)
Psoralen	1.902 ± 0.003
Angelicin	1.506 ± 0.003
Neobavaisoflavone	1.321 ± 0.011
Psoralidin	1.310 ± 0.010
Isobavachalcone	0.736 ± 0.006
Bavachinin	1.623 ± 0.011
Bakuchiol	11.713 ± 0.088

## Supplementary Materials

Supplementary materials can be accessed at: http://www.mdpi.com/1420-3049/21/8/1076/s1.
