# Supplementary material for: Quantitative Analysis of Psoralea corylifolia Linne and its Neuroprotective and Anti-Neuroinflammatory Effects in HT22 Hippocampal Cells and BV-2 Microglia
Source: Molecules. 2016 Aug 17;21(8):1076. doi: 10.3390/molecules21081076 (PMC6274380; doi:10.3390/molecules21081076)
Supplement: Supplementary file 1 [file molecules-21-01076-s001.pdf]

Supplementary Materials: Quantitative Analysis of *Psoralea corylifolia* Linne and Its Neuroprotective and Anti-Neuroinflammatory Effects in HT22 Hippocampal Cells and BV-2 Microglia

Yu Jin Kim, Hye-Sun Lim, Jun Lee and Soo-Jin Jeong

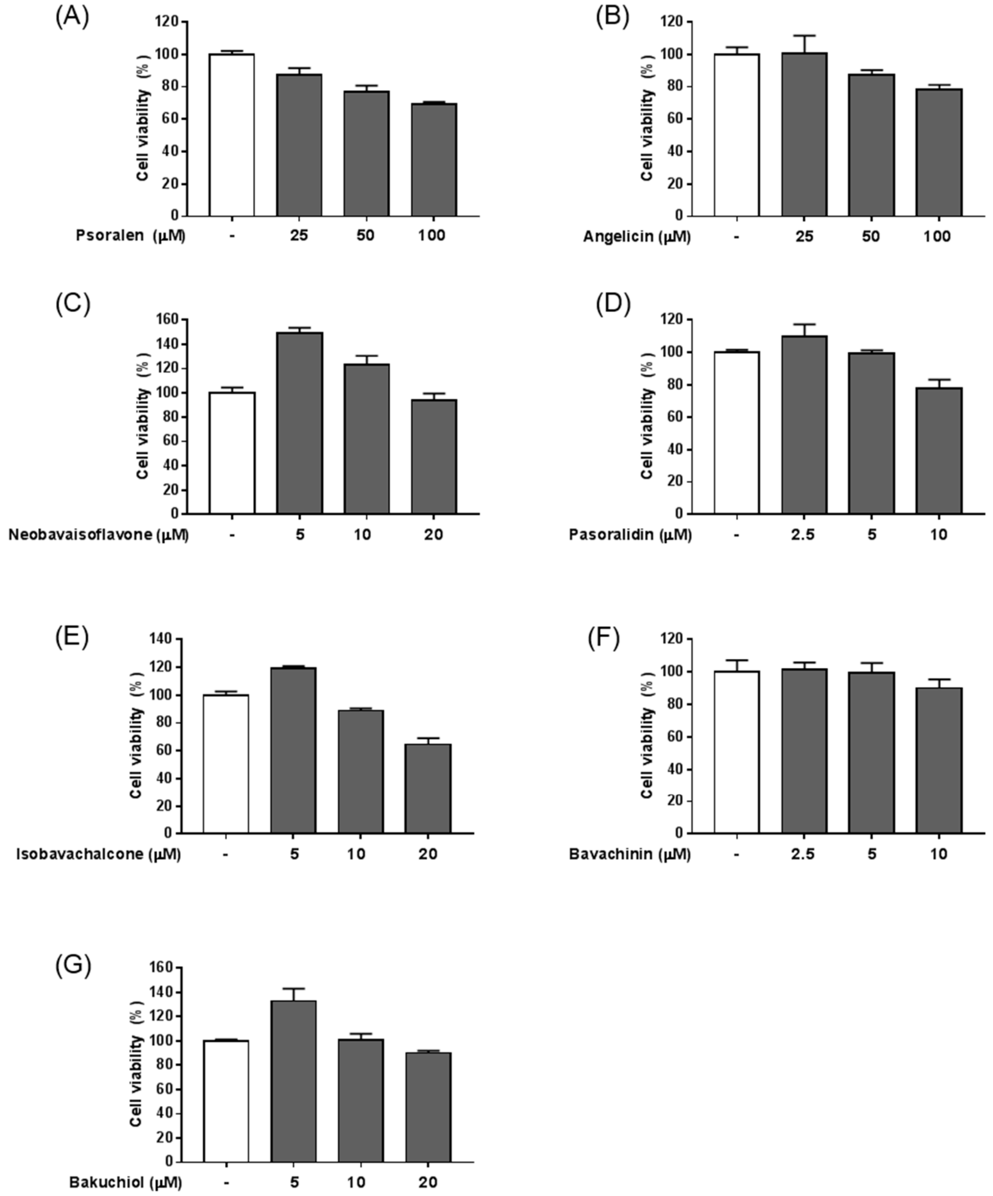

Figure S1. Cytotoxicity of the seven components from *P. corylifolia* in HT22 cells. Cells was seeded onto 96-well plates and treated with various concentration of the seven components for 6 h. Results are shown for psoralen (**A**); angelicin (**B**); neobavaisoflavone (**C**); psoralidin (**D**); isobavachalcone (**E**); bavachinin (**F**); and bakuchiol (**G**). Cell viability (%) was assessed using CCK-8 assay. The value are expressed as the mean ± SEM of three independent experiments.
